# Recent Advances in Non-Conventional Antimicrobial Approaches for Chronic Wound Biofilms: Have We Found the ‘Chink in the Armor’?

**DOI:** 10.3390/biomedicines7020035

**Published:** 2019-04-30

**Authors:** Snehal Kadam, Saptarsi Shai, Aditi Shahane, Karishma S Kaushik

**Affiliations:** 1 Ramalingaswami Re-entry Fellowship, Department of Biotechnology, Pune 411045, India; snehalgkad@gmail.com; 2 Poona College of Pharmacy, Bharati Vidyapeeth Deemed (to be) University, Erandwane, Pune 411038, India; saptarsishai1@gmail.com (S.S.); aditishahane.1998@gmail.com (A.S.)

**Keywords:** biofilm, chronic wounds, antimicrobials, probiotics, pH, nanoparticles, phages

## Abstract

Chronic wounds are a major healthcare burden, with huge public health and economic impact. Microbial infections are the single most important cause of chronic, non-healing wounds. Chronic wound infections typically form biofilms, which are notoriously recalcitrant to conventional antibiotics. This prompts the need for alternative or adjunct ‘anti-biofilm’ approaches, notably those that account for the unique chronic wound biofilm microenvironment. In this review, we discuss the recent advances in non-conventional antimicrobial approaches for chronic wound biofilms, looking beyond standard antibiotic therapies. These non-conventional strategies are discussed under three groups. The first group focuses on treatment approaches that directly kill or inhibit microbes in chronic wound biofilms, using mechanisms or delivery strategies distinct from antibiotics. The second group discusses antimicrobial approaches that modify the biological, chemical or biophysical parameters in the chronic wound microenvironment, which in turn enables the disruption and removal of biofilms. Finally, therapeutic approaches that affect both, biofilm bacteria and microenvironment factors, are discussed. Understanding the advantages and limitations of these recent approaches, their stage of development and role in biofilm management, could lead to new treatment paradigms for chronic wound infections. Towards this end, we discuss the possibility that non-conventional antimicrobial therapeutics and targets could expose the ‘chink in the armor’ of chronic wound biofilms, thereby providing much-needed alternative or adjunct strategies for wound infection management.

## 1. Introduction

### 1.1. Chronic Wounds and Biofilms

Cutaneous wounds result from injury or a break in the epithelial lining of the skin, leading to disturbances in skin anatomy, physiology or function. This breach likely results from an external injury (trauma or surgical incisions) but is often associated with underlying predisposing factors such as diabetes, hypertension, obesity, malignancies, peripheral vascular disease, prolonged immobilization and advanced age [1]. Following this breach in integrity, wounds heal in a well-organized, coordinated process, consisting of four distinct, overlapping phases of haemostasis, inflammation, tissue proliferation and tissue remodelling [2]. These phases typically progress in a predictable manner, resulting in wound healing without the need of significant intervention. Wounds that fail to heal in this manner and instead get arrested in a prolonged inflammatory to proliferative phase, leading to a persistent, non-healing state are defined as chronic wounds [3].

Microbial infections are a leading cause of delayed wound healing, contributing to this chronic wound state [4]. Features of the wound bed, such as an exposed epithelial barrier with devitalized tissue, a moist, nutrient-rich environment and dysregulated inflammatory processes [5], provide a favourable environment for microbial proliferation. Pathogenic microbes, bacteria or fungi, that infect or invade the wound bed typically form biofilms, which are highly structured, three-dimensional microbial aggregates embedded in a self-produced, extracellular polymeric matrix. The biofilm state has been widely reported in chronic wounds (different studies report that 60–90% of chronic wounds contain biofilm-forming bacteria), in fact, current evidence supports the view that biofilms are ubiquitous in chronic wounds [3,6,7].

### 1.2. The Chronic Wound Biofilm Microenvironment

Almost all wounds are initially colonized with commensal flora, which plays a critical role in initiating various inflammatory and signalling events in the wound bed [8]. However, the chronic wound biofilm state results from pathogenic microbes and is most often polymicrobial [8]. Most common bacteria implicated in chronic wound biofilms include *Staphylococcus aureus*, *Pseudomonas aeruginosa* and β-haemolytic Streptococci; other bacteria include *Enterococcus* spp, *Klebsiella pneumoniae*, *Acinetobacter baumanii and Enterobacter* spp. (ESKAPE pathogens), coagulase-negative Staphylococci and *Proteus* spp [8]. While the focus has largely been on the diverse bacterial pathogens in chronic wounds, the role of fungi (particularly *Candida* species) in wound biofilms is assuming significance [9,10]. In biofilms, bacteria form aggregates of microcolonies encased in an extracellular polymeric substance (EPS). These biofilm aggregates are intricately associated with granulation tissue (eschar) in the chronic wound bed [11] and are typically found dispersed among host cells (such as fibroblasts, keratinocytes) and extracellular matrix (ECM) elements (such as collagen, fibronectin, elastin) [12].

Owing to multiple factors, the chronic wound biofilm state displays delayed and defective healing, as well as increased recalcitrance to immune clearance and antimicrobial therapies [13]. In chronic wound granulation tissue, keratinocytes and fibroblasts possess reduced migratory and proliferative capacity, resulting in decreased ECM production and dysregulated inflammatory and antimicrobial responses [14]. On the other hand, the presence of pathogenic biofilm-forming bacteria in the chronic wound bed stimulates a massive influx of immune cells such as neutrophils and macrophages, however their phagocytic, chemotactic and antimicrobial activity is reported to be diminished [15]. As a result, the biofilm burden continues to increase, setting up a vicious cycle of biofilm growth and dysfunctional immune cell infiltration. This results in a sustained pro-inflammatory state, possibly marked by excessive Toll-Like Receptor (TLR) signalling, leading to a massive release of cytokines, chemokines and growth factors [16]. This inflammatory storm also creates a highly proteolytic environment, due to the release of high levels of matrix metalloproteinases (MMPs). While MMPs perform a range of important functions in wound repair, including remodelling ECM components such as collagen and fibronectin, persistently high levels of MMPs degrade newly-formed ECM components, resulting in a state of matrix deficiency. Accumulation and deficient removal of infiltrating immune cells also leads to excessive production of Reactive Oxygen Species (ROS) [17]. In the wound bed, ROS are known to enable antimicrobial activity [18]; however, an excess build-up of ROS creates high oxidative stress in the wound bed. Angiogenesis in the wound bed results in temporary reperfusion that delivers new oxygen to the site, which in turn leads to increased ROS production [19]. This new oxygen is also consumed by the increased influx of immune cells and biofilm bacteria, leading to localized regions with low oxygen tension [20,21]. Localized hypoxia is seen to promote biofilm matrix formation and increase bacterial persistence, contributing to the recalcitrant biofilm state [22,23]. Increased bacterial proliferation also shifts the chronic wound microenvironment to an alkaline pH, which not only has a detrimental effect on host cellular function but possibly promotes biofilm formation, thereby fuelling the prolonged inflammatory-proliferative phase [24]. As a result of the complex interplay between these processes, the chronic wound microenvironment is an alkaline milieu, bathed in exudate rich in pro-inflammatory mediators, degraded ECM components, necrotic cell debris, matrix-degrading enzymes and free radicals [25]. For chronic wound biofilms, this provides an ideal milieu, with stable attachment to host tissue, sustained nutrition, an optimum chemical microenvironment and a background of sustained, unresolved inflammation [26].

It is therefore evident, that the chronic wound microenvironment and biofilm state sustain each other in a highly complex, dynamic and proximate interaction. This has partly been the reason why the management of chronic wound biofilms has been challenging, with increased resilience and intractability to standard approaches of care.

### 1.3. Status Quorum in the Treatment of Chronic Wound Biofilms

Treatment approaches for biofilms in chronic wounds have relied heavily on the use of conventional antibiotics and antimicrobials. These include widely-used topical antibiotics and antiseptics and systemic antibiotics, including broad spectrum agents [27,28]. However, for several reasons, chronic wound biofilms often respond poorly to antibiotic regimens, rendering their extensive use ineffective and even indiscriminate and unnecessary, given the risk of emergence of antibiotic-resistant strains.

As with biofilms in general, chronic wound biofilms are highly tolerant to antibiotics and antimicrobials [29]. This is partially due to the biofilm construct, where the presence of a thick EPS matrix results in poor antibiotic penetration and enzymatic degradation or charge-related neutralization of certain antimicrobials [30]. In addition, intrinsic factors related to the bacteria in the biofilm state, such as reduced metabolic activity, slower growth rates and formation of highly-tolerant persister cells and small-colony variants, also contribute to this state of resistance [31]. Further, as discussed previously, microenvironmental factors such as poor oxygenation, pH variation and increased oxidative stress, could decrease the distribution, availability and thereby the efficacy of antibiotics in the chronic wound bed [32,33]. Given this inherent recalcitrance of chronic wound biofilms to antibiotics, expanding the arsenal of antibiotics or developing novel antibiotic regimens, will have limited effect.

There is therefore the need to develop alternative and/or adjunct ‘anti-biofilm’ strategies, that look beyond standard therapeutic approaches and/or targets. It is also important that such approaches account for the effects of fundamental factors in the chronic wound biofilm microenvironment that contribute towards this state of antibiotic tolerance. In this review, we outline the recent developments in non-conventional antimicrobial approaches to tackle chronic wound biofilms. The first section discusses therapies that directly target biofilm microbes in chronic wounds, using processes or delivery mechanisms distinct from conventional antibiotics. The second section focuses on strategies that modify biological, chemical or biophysical parameters in the chronic wound microenvironment, which in turn enables the disruption and removal of biofilms. Finally, we discuss therapeutics that affect both, biofilm bacteria and microenvironment factors, underlining their multi-pronged targets.

For each section, non-conventional antimicrobial therapies have been selected based on the following criteria (i) novel approaches that have been recently developed or introduced or (ii) recent advances in established therapies. For each treatment strategy, advantages, limitations and current status of the field are summarized in Table 1. Finally, given these advancements in treatment approaches, we address the question: Can the usage of non-conventional antimicrobial therapeutics expose the susceptibility of chronic wound biofilms, thereby improving therapeutic response and clinical outcome?

## 2. Recent Advances in Non-Conventional Antimicrobial Approaches for Chronic Wound Biofilms

### 2.1. Antimicrobial Therapies that Directly Target Microbial Processes

In this section, we focus on non-conventional antimicrobial approaches with mechanisms of action that primarily target microbial structure and/or function. These mechanisms include the direct lysis of the host bacterial cell (as in phage therapy), alterations in bacterial cell membrane permeability, inhibition of respiratory enzyme function and destruction of biofilm matrix (as with metallic, non-metallic and natural product-based nanoantimicrobials), photodynamic inactivation of bacterial metabolism (blue light therapy) and varied mechanisms that target bacterial signalling (quorum sensing inhibitors).

#### 2.1.1. Phage Therapy

Bacteriophages are viruses that are natural predators of bacteria, injecting their DNA into bacterial cells, thereby infecting them. In the case of lytic phages, the infecting phage multiplies to form new virus particles, which are then released by lysis and killing of the bacterial cell [34]. Given this, the use of bacteriophages as antibacterial agents has gained substantial and sustained interest over the years [35,36,37,38].

Bacteriophages have been extensively studied as therapeutic agents for acute burn wound infections, alone or in conjunction with other therapeutics [39,40]. Using both in vivo models and limited clinical trials, phage treatment has demonstrated activity against acute forms of *P. aeruginosa* and *S. aureus*, without any reported adverse effects [41,42,43]. Consequently, bacteriophage therapy against biofilms has received attention [44,45], where it offers potential advantages over conventional antibiotics. A phage’s ability to infect a bacterium is highly dependent on cell surface interactions, leading to a high specificity of bacterial strains a particular phage can infect [46]. This specificity could enable phage-mediated elimination of pathogenic bacteria, while preserving beneficial flora at the site of infection [47]. The high density of bacteria in biofilms also enables the speedy and efficient propagation of phage [48]. Phage infection also induces the production of certain enzymes, such as depolymerase (phage-encoded) and alginase (from the host cell), that break down biofilm matrix elements [49]. Finally, bacteriophages are able to infect persister variants and when these dormant cells switch to growth mode, the infecting phage multiplies to cause cell lysis [48].

Given these advantages, the evaluation of phage therapy for the treatment of biofilms in chronic wounds has received attention, with a large body of published work employing ex vivo or in vivo wound model systems.

Using an ex vivo porcine wound infection model, the effects of phage treatment on *S. aureus* biofilms was evaluated [50]. Inoculated wounds were allowed to grow biofilms for 48 h and then subject to phage treatment at different time points. The number of phages obtained from the infected wounds was higher than the inoculum, indicating the phages underwent replication. After a single phage treatment at 4 h, a reduction in the number and metabolic activity of bacteria was observed. However, a second phage treatment at 48 h, did not result in a significant reduction in bacterial counts, even though metabolic activity remained reduced. This indicates that while phage therapy is effective in the early stages of biofilm formation (possibly when cells are transitioning from planktonic to biofilm state), they are less effective on fully-formed biofilms. Given this, phage treatment regimens would be indicated early in infection, possibly prophylactically and may need to include multiple doses and combination therapies.

To establish efficacy against fully-formed biofilms, it is critical to assess effects of phage therapies in chronic wounds that have progressed beyond the first few hours of infection. For this, in vivo studies have employed multiple dosage protocols and phage cocktails [51,52,53]. In vivo rodent and pig chronic wound models infected with *S. aureus*, *P. aeruginosa* and *Acinetobacter baumanii*, were subject to a cocktail of lytic bacteriophages for up to 8 days post-infection [52]. Results indicated that topically administered bacteriophage decreased bacterial colony counts after 4 days of infection, promoting resolution of the chronic infection. Notably, this study tested phage treatment in combination with wound debridement (as would occur in the standard practice of wound care), which is known to significantly reduce bacterial burden. This is important, as it enables the possibility of using phages in combination with standard approaches of care, to improve overall therapeutic outcome.

In recent work, the effect of combining chestnut honey with phage therapy for *E. coli* and *P. aeruginosa* biofilms was evaluated in an ex vivo porcine skin model [53]. The combinatorial approach showed promising results against 24-h old skin explant biofilms but efficacy was found to vary significantly based on the presence of mono- or multi-species biofilms and time points of treatment for these conditions. Further, maintaining phage viability in the presence of enhancing agents such as honey is a concern, indicating the need for more work into delivery and treatment regimens.

While ex vivo models have provided insights into phage therapy for wound biofilms, they do not enable the study of host immune factors, which could critically influence the efficacy of phage treatment. For example, in an in vivo hind-paw mouse model of diabetic wound infection with methicillin-resistant *S. aureus* (MRSA), combining bacteriophage with linezolid showed a marked reduction in myeloperoxidase (MPO) levels (after 5 days of infection), as compared to either agent alone [40]. The effect likely reduced early bacterial burden, preventing the excessive accumulation of neutrophils and improving tissue healing. This indicates that host immune factors could be leveraged towards developing effective phage regimens and future in vivo work should study the impact on immune parameters.

There are several reports of clinical trials evaluating the usage and safety of phages for infected wounds in humans, mostly focusing on burn and post-surgical infections [43]. Recently, in a randomized phase trial (PhagoBurn), a cocktail of *P. aeruginosa* phages was evaluated for safety and effectiveness in infected burn wound patients. At the low concentrations tested, the cocktail was reported to reduce bacterial burden in infected wounds, albeit slower than the standard of care [41]. However, none of these trials specifically report on the presence of biofilms in these wounds or the efficacy of the phage intervention on the formation of biofilms.

In spite of sustained interest over the years, the use of phages as therapeutic agents has yet to gain a foothold in infection management, except in a few countries [54]. Taken together, the current status of the field clearly indicates that bacteriophages are a promising therapeutic adjunct for the management of chronic wound biofilms. Future studies, in vivo and possible human trials, could focus on studying phage treatment on fully-formed biofilms, analogous to those likely to be seen in clinical practice.

#### 2.1.2. Nano-Based Technologies

The development of a range of nanoantimicrobials-based approaches to reduce antibiotic dependence, augment the effects of current antibiotics and overcome antimicrobial resistance has gained increased significance. Certain nanoparticles have their own intrinsic antimicrobial activity (for example metal-based nanoantimicrobials such as silver, zinc and copper), while others enhance the effect of combination treatments (such as magnetic hyperthermia-based technology that employs iron oxide nanoparticles and d-amino acids). In addition, certain nanoantimicrobial approaches target the biofilm matrix, enabling disruption or improving penetration.

Metallic agents such as silver, zinc and copper, are known to have antimicrobial properties and have been at the forefront in the management of wound infections [55]. Notably, these metals possess bactericidal activity even at nanometric sizes and engineered metallic nanoparticles have been incorporated into a range of wound infection therapeutics [56].

Silver possesses broad-spectrum antimicrobial activity, that has been demonstrated against a wide-range of pathogens, including MRSA and fungi [57]. While the exact mechanism of action is still not completely known, effects appear to be mediated via binding of silver particles to bacterial structures such as the cell wall, cell membrane, DNA and enzymes. Interactions with the cell membrane, result in alterations in nutrient permeability and concentration gradients, which can lead to cell death. Other possible mechanisms include the formation of free radicals and interference with respiratory chain enzymes and all of these mechanisms are likely to be acting together, leading to bacterial cell death [57]. In the context of biofilms, silver has been shown to disrupt the biofilm extracellular polymeric matrix by competing for binding sites [58], thereby destabilizing intermolecular adhesion forces.

Various studies have demonstrated the in vitro and in vivo effects of silver nanoparticles to inhibit both, early biofilm formation and mature biofilms, of wound pathogens [59,60]. In one study, the effect of citrate-capped silver nanoparticles of various sizes, alone and in combination with the antibiotic aztreonam, was evaluated against in vitro, pre-formed *P. aeruginosa* biofilms [61]. Specific combinations of small silver nanoparticles and aztreonam demonstrated synergy in preventing the recovery of 20-h *P. aeruginosa* biofilms following treatment, showing significant defects in biofilm architecture. This indicates the potential for silver nanoparticle-based therapies to be employed in conjunction with antibiotics, whereby it could potentially reduce the dosage and duration of antibiotic treatment.

In an in vivo study, silver nanoparticles coated with polyethylene glycol (PEG) were loaded onto hydrogels and evaluated for their efficacy using a MRSA mouse wound infection model [62]. Hydrogels with PEG-coated silver nanoparticles demonstrated consistent bacterial load reduction over 15 days, which was superior in time and effect to that seen with standard silver sulfadiazine cream. In addition to its antimicrobial effects, treated groups also showed healing of epidermal layers and restoration of stratification.

Silver nanoparticles have also been shown to promote other wound healing parameters such as fibroblast migration and macrophage activation, holding potential to extend its benefits beyond biofilm management in chronic wounds [63].

Other metallic nanoparticles, such as zinc and copper, have also been explored for their antimicrobial applications for wound infections. As with silver, their exact mechanisms of action are not fully elucidated but are believed to be via interactions with cell membrane structure, leading to loss of membrane integrity and bacterial death. Other possible mechanisms include production of reactive oxygen species, lipid peroxidation, protein oxidation and DNA damage [57].

Using in vitro and in vivo approaches [64], ZnO nanoparticles were determined to be effective in reducing superficial and deep *S. aureus* bacterial load in a mouse wound infection model. Effects were observed as early as 7 days post infection and after 21 days the effect of ZnO nanoparticles was equivalent to that of the antibiotic tetracycline. In another study, an experimental purulent rat wound MRSA model was treated with a combined suspension of zinc and copper nanoparticles and chitosan [65]. The combination proved to be effective in rapidly eliminating the MRSA wound infection, which started as early as day 3 and was completed by day 7. It is also important to note that, even though various forms of silver have been in use for decades, there has been only a few reports of bacterial resistance [66]. While this could change with extensive use, a report on antimicrobial resistance to silver nanoparticles indicates that mechanisms that drive resistance are likely phenotypic and reversible [67].

Another interesting nano-based system, based on magnetic hyperthermia, uses the heating potential of iron oxide nanoparticles when exposed to an alternating current magnetic field. This has been shown to possess anti-biofilm properties for a wide-range of bacterial pathogens [68,69,70,71]. A recent therapeutic approach combined magnetic hyperthermia with d-amino acids, know to inhibit and disperse biofilms [72]. Using a novel two-step treatment, a pre-incubation step allows the d-amino acids to initially disrupt the biofilm EPS, followed by exposure to an alternating magnetic field. This combination treatment resulted in complete eradication of in vitro *S. aureus* biofilms. Any remaining bacterial cells post treatment were not viable, indicating that this treatment would prevent cells from reattaching and establishing a new biofilm. The magnetic field treatment parameters and incubation with d-amino acid did not adversely affect mammalian cells. Given the complete biofilm eradication observed as well as the low cytotoxicity of this method, further in vivo studies are warranted.

Nano-based approaches for wound infection treatments are not only limited to the use of engineered nanoparticles. Recent work employed nanoscale features to design nanohybrid enzymes or nanozymes to target wound infection biofilms [73]. The hybrid nanozyme combines gold nanoparticles and ultrathin graphitic carbon nitride, to activate biologically relevant levels of ROS. ROS are known to possess antimicrobial activity but they exhibit cytotoxicity at high concentrations, which limits their therapeutic applications [74]. Following treatment with H_2_O_2_, ultrathin graphitic carbon nitride exerts a peroxidase-like activity, which catalyses the decomposition of H_2_O_2_ to •OH radicals, with potent antibacterial activity. The presence of gold nanoparticles helps stabilize these free radicals, allowing the use of smaller amounts of H_2_O_2_. This strategy showed almost complete eradication of in vitro *S. aureus* biofilms, with observable cellular deformations and breach in cell adhesions. When evaluated using mouse wound infection model, a band-aid formulation with this nanozyme could prevent wound infection and accelerate wound healing. In addition to the possibility of external application of this ROS-nanozyme formulation, this strategy could harness the effects of biological ROS. Given the presence and critical role of ROS in chronic wound infections, nanohybrid enzymes could potentially be leveraged to fine-tune the antibacterial activity of native ROS, while also preventing prooxidant damage.

Various natural products and their derivatives also exhibit anti-biofilm activities and notably, can exert these effects via non-lethal mechanisms [75,76]. In recent work, chitosan encapsulated ferulic acid nanoparticles were evaluated for their efficacy to treat biofilms in wounds [77]. Ferulic acid is a plant derivative that is known to possess antibiofilm activity against *Candida albicans* [78]; however, due to stability and permeability issues, its therapeutic use is limited. Nano-encapsulation of ferulic acid in chitosan circumvents these limitations. The small size and high surface area to volume ratio of nanoparticles, enables easier biofilm penetration. Further, chitosan is biocompatible, biodegradable and relatively non-toxic. Chitosan encapsulated ferulic acid nanoparticles significantly reduced the metabolic activity of *Candida* biofilms, with. scanning electron microscopy revealing distorted cellular morphologies. Further, concentrations of ferulic acid used to generate nanoparticles were demonstrated to be non-toxic to human keratinocyte cell lines. Interestingly, ferulic acid has shown to have antimicrobial properties against *S. aureus* as well [78], broadening its potential as a therapeutic agent.

Together, these results indicate that nanoantimicrobials are a very promising approach in the management of wound biofilms. As more healthcare and consumer products with impregnated nanoparticles become available, this approach holds potential to reduce the overuse of conventional antibiotics, while still targeting microbes directly.

#### 2.1.3. Blue Light Therapy

The use of light therapy, both in the infrared and visible spectrum, positively impacts wound healing by increasing host cell proliferation, angiogenesis, granulation tissue formation and collagen synthesis [79,80]. In recent years, visible light, particularly blue light (wavelength 400–500 nm), has been studied for its antimicrobial and antibiofilm effects [81,82]. While the exact mechanisms involved are not fully understood, photodynamic activation of microbial porphyrins or photosensitive dyes, leading to the production of toxic reactive oxygen species (ROS), has been proposed [83]. In one large in vitro study, preformed biofilms (72 h old) of a range of nosocomial wound pathogens including *P. aeruginosa, S. aureus, A. baumannii* and *K. pneumoniae,* were exposed to different intensities of blue light for different time periods [84]. All biofilms tested demonstrated high susceptibilities, with significant reduction in viability. Notably, in this study, Gram positive biofilms such as *S. aureus* were much less susceptible as compared with Gram negative biofilms, possibly due to the production of light-protective bacterial pigments. This variability in response underscores the importance of testing blue light on multi-species biofilms (as found in chronic wounds), given that several bacteria produce pigments as virulence factors.

One approach to employing blue light for mixed species biofilms, particularly those with Gram positive pathogens, is using it in conjunction with photosensitizing, nontoxic dyes. These dyes, when activated by low level visible light result in the production of ROS and cell death (photodynamic therapy) [85,86,87].

Also relevant in the context of polymicrobial chronic wound biofilms, is the less-studied effect of blue light on fungal biofilms [88]. Following inoculation with *C. albicans*, murine skin wounds were subjected to blue light irradiation once a day for three days. Blue light therapy was observed to induce the killing of *C. albicans* in the biofilms, with almost no viable cells by the final day of treatment. Notably, no adverse effects were reported when human skin fibroblasts and keratinocytes were exposed to a similar dosage regimen. While further work is needed on its efficacy on fungal biofilms, blue light clearly holds potential to target both bacterial and fungal elements in chronic wound biofilms, which is a distinct advantage over conventional antibiotics and even most non-conventional approaches.

A large number of preclinical in vivo animal studies have established the efficacy of blue light on biofilms of *P. aeruginosa, S. aureus* and *A. baumannii* in chronic wounds [89,90,91]. While a reduction in bacterial counts and virulence was observed, it is important to note that treatment was initiated very soon after bacterial inoculation (as early as 30 min); while this is possible in experimental systems, this is less applicable in the clinical scenario. Given this, future in vivo animal studies could focus on the effect of blue light on older biofilms and possibly in conjunction with standard approaches of care.

Based on this work, blue light therapy, alone or in combination, stands as a promising approach to decrease the bacterial burden of biofilms with promising in vitro and in vivo results. A low potential for tolerance development, activity against a wide-range of wound pathogens, ease of administration, minimal observed adverse effects to host cells, are all factors that support its further exploration [92]. In addition to its antimicrobial effect, blue light has also been reported to modify the structure of the biofilm matrix [93], an effect that could be further explored to enhance the susceptibility of chronic wound biofilms.

#### 2.1.4. Quorum Sensing Inhibitors

During the process of biofilm formation, bacteria communicate with each other using quorum sensing (QS) circuits via cognate receptors and signal molecules [94,95]. Disabling these QS circuits with small molecules is a potential strategy to prevent biofilm formation and proliferation, particularly for wound pathogens *P. aeruginosa* and *S. aureus* [96,97]. Several natural and synthetic QS inhibitors have been identified and evaluated for their usage as antibacterial strategies [98,99,100,101], with varied mechanisms of action such as degradation of signal molecules, inhibiting signal synthesis or binding or blocking the signal transduction cascade [102,103]. In chronic wound biofilm models, QS inhibitors have been able to reduce colonization, biofilm formation and virulence of *P. aeruginosa* and *S. aureus* [104].

In a mouse wound model of *P. aeruginosa* infection, chlorogenic acid was observed to inhibit biofilm formation, decrease bacterial load and accelerate wound healing [105]. This was determined to be via the downregulation of *P. aeruginosa* QS-related receptors (LasR, RhlR and PqsR), referred to as quorum quenching. As predicted via computational modelling, chlorogenic acid could form hydrogen bonds with these three QS receptors, which could inhibit the binding of the native inducers. Further, using a *C. elegans* based model of *P. aeruginosa* infection, treatment with chlorogenic acid was also seen to reduce bacterial burden and extend the survival period of the infected nematodes. This is important, given that there is a large overlap between *P. aeruginosa* virulence factors needed for nematode killing and pathogenesis in humans.

Quorum sensing in *S. aureus* has been demonstrated to be inhibited by RNAIII inhibiting peptide (RIP) [106,107] and RIP derivatives have been explored to treat biofilm-associated infections [108]. A recent study used one such derivative, FS10 [109], in conjugation with tigecycline in an in vivo *S. aureus* wound infection model. For the 8-day old infected wounds, including those with MRSA, the combination demonstrated a synergistic effect, with a greater reduction in bacterial numbers and better wound healing parameters. QS inhibitors against *P. aeruginosa* have also demonstrated a marked synergistic effect in conjunction with antibiotics such as tobramycin, including in in vitro wound biofilm models [109,110,111].

While QS molecules have primarily been implicated in biofilm formation in non-healing wounds, *P. aeruginosa* autoinducers have also been shown to interfere with host cell signalling [112,113]. For example, the autoinducer 3OC12-HSL induces the expression of MMP-9 in rat fibroblasts and could possibly contribute to wound chronicity. Therefore, using QS inhibitors to target bacterial virulence and pathogenesis, could serve as a multi-pronged approach in the management of chronic, non-healing wounds.

A major concern with the use of QS inhibitors has been their toxic effects on host cells, however, certain inhibitors have demonstrated none or limited toxicity to mammalian cells at working concentrations [111]. Also, while QS inhibitors have shown efficacy in vitro, often their efficacy in more complex, in vivo model systems is reduced [114]. Further, their biofilm-inhibition effects are highly strain-dependent, particularly for clinical isolates. This also raises the need to better understand the role of QS inhibitors in polymicrobial wound infections. These have been some of the limiting factors in taking QS inhibitors to clinical trials [104] and further work would need to focus on developing appropriate model systems that serve as a bridge between promising in vitro results and anticipated clinical trials.

### 2.2. Antimicrobial Therapies that Target the Chronic Wound Biofilm Microenvironment, Indirectly Affecting Microbial Growth and Survival

In this section, we focus on non-conventional antimicrobial approaches that modify various factors in the wound biofilm microenvironment, which in turn results in microbial killing and/or inhibition. These mechanisms include modification of local pH (by application of external treatments or using native by-products of microbial metabolism), removal of exudate (as with negative pressure wound therapy and surfactants), promotion of granulation tissue formation and angiogenesis (with hyperbaric oxygen therapy) and production of local ROS (bioelectric dressings).

#### 2.2.1. pH Modulation

It is well established that the pH of the wound bed has a critical role to play in the persistent, non-healing wound infection state [32]. Several components of the chronic wound infection microenvironment are affected by pH changes including angiogenesis, collagen formation, activity of MMPs and immune cell function. The chronic wound bed pH typically exists in the alkaline range (7.15–8.9) [115], resulting partly from the production of basic by-products of bacterial proliferation. In turn, these changes in pH affect microbial density and composition in the infected wound [24,116]. For example, an alkaline shift in pH has been shown to increase the density of biofilms, possibly related to changes in the growth rate of bacteria [117]. In addition, an alkaline pH results in reduced local oxygen release, thereby promoting the growth of anaerobic bacteria [116]. Given this, targeting the pH of the wound bed has been explored as a strategy to combat wound biofilms and thereby pushing the wound towards resolution of the non-healing state. One approach uses acidic treatments such as acetic acid (1% and 5%), citric acid, boric acid and ascorbic acid as well as Manuka honey [118], have been evaluated for their effects towards reducing wound bed pH and anti-biofilm activity [119,120,121]. Applying agents that lower wound bed pH, has been shown to inhibit the growth and multiplication of bacteria and reduce the toxicity of bacterial end products such as ammonia [122]. While acetic acid has shown promising in vitro results and in the clinical setting, it has particularly been effective only against *P. aeruginosa* biofilms [121]. This poses a limitation in the context of polymicrobial infections, which are often encountered in chronic wounds. However, other agents such as citric acid have shown beneficial results with chronic wounds infected with a range of pathogens including *S. aureus* [122]. Also, important is the fact that most therapies for wound infections, including antibiotics and enzymatic treatments, have an optimum pH at which they exert their effects [117]. For example, in the presence of an acidic milieu the activity of certain fluoroquinolones is enhanced [116]. However, certain antibiotics the fluoroquinolone ciprofloxacin and macrolides demonstrate a loss of activity at acidic pH [32]. On the other hand, an alkaline pH has been shown to increase susceptibility to aminoglycosides including tobramycin, widely used against *P. aeruginosa* [123]. Notably, the bacterial metabolic by-product of amino acid metabolism, possibly ammonia or amines, caused a shift towards alkaline pH that restored the susceptibility of planktonically-grown antibiotic-resistant strains. It is tempting to speculate that the alkaline pH of chronic wounds could be harnessed to enhance such antibiotic effects. However, when the effect of combining the alkali bicarbonate with tobramycin was tested against in vitro biofilms, the effect was determined to be antagonistic, actually promoting biofilm growth [183]. Hence, given the varied effects of pH on different components of the wound infection microenvironment, it remains to be seen how pH modulation can present a comprehensive solution to target infection control, wound healing and improve treatment efficacy.

#### 2.2.2. Negative Pressure Wound Therapy

Negative Pressure Wound Therapy (NPWT) or vacuum assisted wound closure is a standard of care in wound management, in which continuous or intermittent sub-atmospheric pressure is applied to the surface of the wound. NPWT systems typically consist of a polyurethane ether foam sponge, semi-occlusive adhesive cover, fluid collection system and a suction pump [124]. Using this apparatus, the contact filler (such as polyurethane) enables the applied negative pressure to reach the wound bed, facilitated by an air tight vacuum seal. NPWT targets several key factors in the chronic wound infection microenvironment such as decreasing the accumulation of exudate, enhancing granulation tissue and blood vessel formation, improving oxygen and nutrient supply, which possibly contribute to its antimicrobial effects. The effectiveness of NPWT as an antimicrobial approach has been studied, particularly in conjunction with wound instillation [125,126]. In recent work, the effects of NPWT in conjunction with automated wound antiseptic irrigation was investigated on 111 patients with infected wounds [127]. NPWT with instillation showed a significant reduction in the bacterial contamination load and number of different bacteria for a range of infected wound types. Notably, the study did not characterize the extent and nature of biofilm formation in the infected wounds, which would be likely to affect treatment outcomes. On the other hand, NPWT with acetic acid instillation has shown promising results in infected wounds with mature biofilms. For a series of patients, NPWT with acetic acid significantly reduced mucoid infection with *P. aeruginosa*, as evidenced by clinical photographs [128]. Further, this combination was shown to clear pathogenic microbes in highly recalcitrant wound biofilms [129]. Other parameters indicating the resolution of chronic inflammation, such as a decrease in the MMP-9/TIMPS ratio, increase in MMP-1, increase in procollagen levels, increase in VEGFR expression corresponding to enhanced tissue vascularization and proliferation of fibroblasts and keratinocytes, were also observed. Given that NPWT is widely used in the management of non-healing wounds, it presents an ideal technique to be used in combination with topical antiseptic instillation, towards combination approaches for chronic wound infections.

#### 2.2.3. Hyperbaric Oxygen Therapy

Hyperbaric Oxygen therapy or HBOT, a technique evolved in the past 40 years, has been proposed as an adjunct therapy for chronic wounds [130]. It is well-established that in their phase of arrest, chronic wounds are in state of perpetual hypoxia, which limits wound healing [21,26,131]. In HBOT, 100% oxygen, at higher than atmospheric pressure, is supplied to the peripheral tissues and skin for a defined period of time, resulting in hyperoxia. The treatment usually involves systemic inhalation in enclosed vessels capable of raising the partial pressure of oxygen. While there has been controversy surrounding its use, recent research has provided more support of its beneficial effects in chronic wounds [132]. HBOT increases the partial pressure of oxygen in the wound bed, leading to the production of reactive oxygen species (ROS) and reactive nitrogen species (RNS). These promote wound healing by activating local growth factors, promoting angiogenesis and increasing extracellular matrix deposition [133]. HBOT is successfully used for the management of a wide-range of non-healing wounds, such as diabetic foot ulcers and those resulting from venous and arterial insufficiency.

In the context of chronic wound infections, HBOT has been shown to possess antimicrobial activity and has been evaluated for its ability to eradicate biofilm formation [134]. HBOT is believed to exert its antimicrobial effects both, directly via oxidative stress and indirectly via the host immune factors. In addition, HBOT has been reported to act synergistically with certain antibiotics, thereby enhancing their effects [132,135,136,137]. This is significant, given that in the clinical setting, HBOT is commonly administered along with antibiotics for chronic wound infections. This synergistic effect is particularly seen with antibiotics such as beta-lactams, quinolones and aminoglycosides, which mediate at least some of the effects through ROS production and resultant toxicity.

The efficacy of HBOT against biofilm infections has been evaluated using in vitro and in vivo platforms. In one study, polymicrobial fully-formed biofilms (*P. aeruginosa*, *S. aureus* and *E. faecalis*) were subject to HBOT at 30 min intervals. Results revealed small but significant decreases in cell viability at 60 and 90 min intervals [134]; however, a rebound in overall cell viability in the control and treated groups was seen at 90 min. Notably, these in vitro biofilms did not receive any additional therapies, which in a clinical scenario would be part of the standard of care. Therefore, in vivo studies that test HBOT in conjunction with standard therapeutics would be important. The same study recruited patients with chronic, non-healing wounds and subjected them to HBOT treatments, in conjunction with standard of care such as cleansing and debridement [134]. Adjunct treatment with HBOT was seen to reduce bacterial load as compared with standard of care alone. Using 16S rDNA sequencing, marked changes in species composition were observed during the duration of HBOT applications. This indicates complete disruption and reorganization of the biofilm community, leading to greater microbial diversity and possibly better responses to treatment.

While it is clear that its role as an adjuvant to standard wound infection therapeutics holds potential, further evaluation of HBOT with different antibiotics and for different biofilm stages is needed [138]. Though not equivalent to HBOT, topical oxygen delivery devices have also shown promising results for infected wounds and further evaluation of their applicability and outcome might enable their widespread use [130,131], given the cumbersome nature of HBOT administration.

#### 2.2.4. Surfactants

Surfactants work by reducing surface tension between liquids and a surface, making molecules less likely to stick together. Surfactants play an important role in wound care, where they interfere with the potential for microbes to adhere to the wound surface, thereby reducing chances of infection [139]. Once the surface tension is reduced between the liquid (any liquid used to rinse wounds) and the surface (the wound bed), the liquid (for example, an antimicrobial agent or cleansing solution) can infiltrate the surface and carry away debrided cells and microbes. An FDA approved surfactant polymer dressing (SPD) used for removal of necrotic wound tissue, was tested for its antibiofilm effects [140]. It was found to inhibit aggregation and EPS matrix formation in in vitro *P. aeruginosa* and *S. aureus* cultures, lowering the chances of biofilm formation. When used in combination with antibiotics, a synergistic effect was observed, likely through SPD disrupting EPS matrix, converting biofilms into a more planktonic-like phenotype, which can then be cleared by the antibiotic. Another poloxamer surfactant-based dressing was tested against mature 3-day old biofilms in an ex vivo porcine skin model [141]. Here, porcine skin wounds were inoculated with *P. aeruginosa* and incubated for 3 days for biofilm development. In order to mimic treatment regimes in a clinical setting, the infected wounds were exposed for 24 h to a surfactant-based gel covered with a secondary dressing. A 3-day treatment was followed, wherein after 24 h the gel was wiped off and reapplied during a dressing change (to mimic clinical protocols of dressing changes in wounds). The gel-treated wounds showed a decrease in the biofilm bacteria, while the numbers kept rising in the control wounds (whose treatment was wiping only, with no gel application). Here as well, the authors hypothesize that the effect is mediated by a conversion of biofilm into more planktonic-like phenotype, allowing for easier removal when wiping.

The use of surfactant based wound dressings is a well-established component of wound care management. Given that it is already used in the clinic, exploring its potential role towards the management of recalcitrant biofilms would be valuable. In doing so, surfactant-based antimicrobial approaches could help to disrupt recalcitrant biofilms, as well as increase the efficacy of conventional antibiotic treatment.

#### 2.2.5. Electrical and Electrochemical Approaches

Electric and electrochemical approaches in wound dressings are an emerging area of potential therapeutics [142,143]. A recent study showed the ability of a wireless silver and zinc bioelectric wound dressing (WED) to reduce co-aggregation of *P. aeruginosa* [144]. Biofilm thickness was reduced, and live/dead staining revealed bacterial cell death in WED treated biofilms. While the exact mechanisms are not known, multiple microenvironmental effects such as production of superoxide radicals, inhibition of quorum sensing genes and enhanced keratinocyte migration, are likely involved. The same group used the WED to treat infected wounds in a porcine burn wound model [145]. They observed disruption of *P. aeruginosa* and *A. baumannii* mixed biofilms both at initial stages of infection as well as when applied to mature biofilms.

Electronic scaffolds (e-scaffolds) which generate small amounts of H_2_O_2_ also exhibit antibiofilm properties [146,147]. Within a wound, a polarized electrode converts the oxygen in the wound microenvironment into H_2_O_2_. A small amount of H_2_O_2_ is produced within the wound naturally as well but at higher concentrations it can be cytotoxic to mammalian cells. A carbon-based conductive fabric was used, and the potential required for sustained H_2_O_2_ production at lower quantities was identified [146]. When this polarized fabric was laid on top of existing *A. baumannii* biofilms, the surface coverage of the biofilms reduced, as compared to the control. This e-scaffold also reduced CFU from *A. baumannii* biofilms in an ex vivo porcine skin model. The porcine host tissue showed 95% viability post exposure to the e-scaffold, indicating no significant damage to the underlying tissue cells. A follow-up study by the same group [147] identified that the use of a hyperosmotic agent (Maltodextrin), which could not only cause oxidative damage via gene expression changes but also increase H_2_O_2_ uptake by bacterial cells, thereby increasing the efficacy of the e-scaffold against *A. baumannii* and *S. aureus* biofilms.

Electrochemical dressings have been shown to assist the wound healing process and recently there has been a substantial interest in leveraging them as antimicrobial strategies. While the field it still in a relatively nascent stage, there is a reasonable body of evidence indicating the potential of electric wound dressings to serve as non-antibiotic approaches for wound biofilm management.

### 2.3. Antimicrobial Therapies that Target Bacteria and the Chronic Wound Biofilm Microenvironment, Both Directly and Indirectly Impacting Microbial Growth and Survival

This section focuses on non-conventional antimicrobial approaches that can be leveraged to exert a dual-pronged approach towards the management of chronic wound infection biofilms. Unlike previously described approaches, that directly target bacteria or primarily modify the microenvironment, these strategies have multiple effects on various factors in in the chronic wound infection microenvironment. For example, probiotics act to directly compete with pathogenic bacteria for nutrients and produce antimicrobial compounds but also recalibrate the host immune cellular response and promote host cell proliferation. Another dual-pronged approach, mesenchymal stem cells, exerts direct antimicrobial effects via the production of antimicrobial peptides as well as modulates the function of host immune cells, including the production of inflammatory mediators.

#### 2.3.1. Probiotics

The potential therapeutic effects of commensal probiotics for several pathological conditions is well-established [148] and their potential role in the treatment of chronic wound infections is an emerging area [149]. This approach makes use of non-pathogenic bacterial strains, which can work to counteract the proliferation of pathogenic species and promote restoration of commensal wound microflora. There are several mechanisms by which probiotics are believed to target chronic wound biofilm bacteria, including preventing the attachment and binding of pathogenic bacteria, competing for nutrients, producing antimicrobial compounds (for example bacteriocins), restoring the presence of anti-inflammatory mediators, promoting host cell proliferation and lowering the pH of the wound bed. Together, these effects enable a reduction in the burden of pathogenic species, allowing the re-establishment and proliferation of the normal wound flora. This plays a critical role in recalibrating the chronic wound immune response, towards resolving the prolonged inflammatory-proliferative phase [150]. *Lactobacillus* species are well-established for their probiotic properties and have also been identified as potential probiotics for chronic wound infections [149,151,152,153,154,155]. Several studies have demonstrated the effects of cell-free extracts of *Lactobacillus* as anti-biofilm agents for wound pathogens [156,157]. For example, a cell-free extract of a *Lactobacillus plantarum* strain (F-10) was shown to inhibit in vitro cell growth and biofilm formation in *Pseudomonas aeruginosa* (PAO1), methicillin resistant *Staphylococcus aureus* (ATCC 43300) and certain hospital-derived strains [158]. Upon neutralization of the extract with an alkali, cell growth and biofilm formation were no longer inhibited as efficiently, indicating that the low pH of the extract could play a role in these effects. This study did not elaborate on the exact mechanisms involved in decreased biofilm formation but inhibition of relevant quorum sensing-mediated factors such as motility and rhamnolipid production was observed. Another critical factor in initial biofilm formation in chronic wounds is bacterial attachment to the wound matrix [16] and intercepting early cell attachment is an important anti-biofilm strategy. Using cell-free culture supernatants of a mixture of probiotic strains (*Lactobacillus, Propioniferax, Bifidobacterium*) [159], a decrease in cell attachment, assayed by crystal violet staining, was observed for *S. aureus* and *P. aeruginosa*. Another study evaluated the ability of probiotics to breakdown already-formed biofilms. Supernatants of *Lactobacillus* strains isolated from local milk and yoghurt [160] were able to completely inhibit in vitro biofilm formation when co-incubated with *P. aeruginosa*. Further, when *P. aeruginosa* when applied to overnight biofilms, the extracts were able to remove the previously formed biofilms. While the study did not elucidate the underlying mechanism involved in the disruption of pre-formed biofilms, the *Lactobacillus* strains that showed this effect were also robust biofilm formers themselves, possibly indicating a competition for nutrients or physical space or both.

In vivo models have also established the efficacy of local probiotic therapy with *Lactobacillus* strains, as injections or dressings, in reducing the mortality of wound infections and preventing the dissemination of bacteria from the wound [161,162,163,164].

In addition to their ability to inhibit biofilm formation, probiotic *Lactobacillus rhamnosus* GG lysates have been shown to improve wound re-epithelialization by enhancing keratinocyte recruitment [165]. The mechanism underlying this involved increased expression of the chemokine CXCL2, that accelerates keratinocyte migration and proliferation. Recent work took this further, by combining the local delivery of chemokines with the pH-lowering effects of *Lactobacillus*. Using a plasmid-encoded *Lactobacillus reuteri* strain, the chemokine CXCL12 was delivered locally to mice and human skin wound models [166]. Bacteria-produced lactic acid reduced the wound bed pH, potentiating the effects and bioavailability of CXCL12, which in turn enhanced host cell proliferation, macrophage-induced healing and synthesis of certain ECM components. While the study did not directly test the effects of these changes on pathogen colonization and biofilm formation, these microenvironmental factors are known to influence chronic wound biofilms.

It is clearly evident that probiotic therapy for wound biofilms is an area of increased interest, with a reasonable body of in vitro and animal studies demonstrating that it prevents the early stages of biofilm formation. To understand its efficacy against chronic wounds, the next set of evaluations would need to focus on fully-formed biofilms in in vivo studies and even clinical wounds. Given that probiotics could have a broad spectrum of activity, unlikely to result in antibiotic resistance, can exert beneficial effects even on host tissue and are inexpensive, further work holds promise towards including probiotics in chronic wound biofilm management. The fact that probiotics are being used in clinical and home-based setting for other clinical conditions, would also help override any ethical issues and the notion of it being counterintuitive to add more bacteria to treat a wound infection.

#### 2.3.2. Mesenchymal Stem Cells

Mesenchymal stem cells or stromal cells (MSCs) are self-renewing multipotent cells found in numerous locations within the body, such as bone marrow, adipose tissue, endometrium and placenta [167,168]. MSCs are known to possess antimicrobial activity, which along with their feasibility for in vitro expansion [169,170], has prompted their study as therapeutic agents for infections [171,172]. MSCs exert their antimicrobial effects through direct mechanisms, via the secretion of antimicrobial peptides or via indirect mechanisms that regulate the host immune response [173]. MSC-derived antimicrobial peptides act directly on bacterial surface and intracellular molecular targets and include cathelicidin (LL-37), defensins and lipocalins [171,173,174]. The indirect antimicrobial effects of MSCs, partly mediated via toll-like receptor (TLR) signalling, shown to modulate proinflammatory cytokine and chemokine induction, release immunosuppressive factors that inhibit excessive proliferation and infiltration of inflammatory T cells and natural killer cells and increase phagocytic activity of monocytes and neutrophils. Further, independent of their antimicrobial effects, MSCs have been shown to enhance wound healing by promoting angiogenesis, host cell differentiation and migration and reduce fibrosis and scar formation [175]. Together this indicates, that MSCs could be promising tool in the management of chronic wound infections, where they could target bacteria as well as promote resolution of the persistent inflammatory state.

Possibly the most compelling evidence so far supporting the use of MSCs for chronic wound infections is a therapeutic approach that combines MSC therapy with antibiotics, tested in mouse and dog models [176]. Using a murine wound biofilm model with a surgical mesh coated with *S. aureus*, MSC treatments were administered via tail vein injections. A significant reduction in the biofilm burden at the wound site was observed only in mice treated with antibiotics along with MSCs pre-activated with TLR3 agonists. These results indicated a strong interaction between activated MSCs and antibiotics. When explored further, it was determined that MSC secreted factors, including the antimicrobial peptide cathelicidin, could enhance the bactericidal activity of a range of antibiotics. In addition to these direct effects, MSCs were also observed to stimulate host innate immune factors associated with accelerated wound healing, such as neutrophil phagocytic activity, monocyte migration and phenotypic switching of macrophages to the M2 type. The work was further extended to a spontaneously developed multi-drug resistant (MDR) chronic foot wound infections in a pet dog. After multiple infusions of canine-derived MSCs over 6 weeks, a progressive clearance of MDR *E. coli* and *P. aeruginosa* was seen from the wound bed. This work opens the possibility that the systemic administration of MSCs for chronic wound infections in humans could be a feasible option.

Other reports on the potential use of MSCs for wound infections, have reported efficacy against a range of would-relevant pathogens [177,178,179,180], demonstrating enhanced bacterial clearance and, in the case of in vivo models, a reduction in the pro-inflammatory response and severity of tissue injury. In one study with *S. aureus* and *P. aeruginosa*, MSCs were observed to attach to the surface of *S. aureus* and engulf them. In addition, conditioned medium, harvested from MSC cultures exposed to bacteria, was seen to inhibit *P. aeruginosa* biofilm formation [181]. As evident, MSCs exert their antimicrobial effects via their innate phagocytic activity as well as via the secretion of antimicrobial factors. Though these results are promising, it is clearly evident that more in vivo and human studies are needed to establish the role of MSCs in chronic wound infection management, particularly testing their effects directly on biofilms. In this context, it would also be important to establish appropriate delivery and preconditioning methods for MSCs applied to chronic wound infections; which may include bioengineered skin grafts or priming MSCs with bacterial components such as endotoxin (LPS), TLR agonists or cytokines to enhance their survival and biological effects [175,182].

## 3. Conclusions

It is clearly evident that there is a plethora of research that supports the development and potential use of non-conventional antimicrobial therapeutics for the management of chronic wound infections. In most instances, these therapies will serve as valuable adjuncts to current wound infection management approaches, for example, the usage of bacteriophages, surfactants and probiotics along with current antibiotics. However, in a few cases, these non-conventional strategies also hold potential to offer new treatment paradigms. Possibly the most exciting prospect in this regard is the use of nanoantimicrobials, of which several commercial products are available and in use in the clinic. In general, to explore these possibilities, it would be critical to evaluate these approaches on in vivo platforms and in clinical studies, both alone and in combination with conventional agents. At the same time, select promising approaches should be evaluated for practical considerations such as appropriate delivery mechanisms and treatment regimens. This will serve as a huge step towards introducing and establishing these non-conventional approaches in standard of care practices. In conclusion, this review underlines the fact that non-conventional antimicrobial therapeutics have managed to identify chinks in the biofilm armor and hold tremendous promise in the future management of chronic wound biofilms.

## Figures and Tables

**Table 1:** Summary of non-conventional antimicrobial approaches for chronic wound biofilms.

Therapy	Advantages	Limitations	Current Status of the Therapeutic in Wound Infection Management	References
**Antimicrobial therapies that directly target microbial processes**				
Phage therapy	-Highly-specificity for bacterial strains -High-density biofilms could enable -Efficient propagation of phage -Less likelihood of resistance development -Able to infect dormant cells and persister variants	-Maintaining phage viability in the delivery vehicle is a concern -Phage therapy to gain a foothold in infection management	-Several clinical trials conducted for usage and safety in burns and post-surgical infections	[34,35,36,37,38,39,40,41,42,43,44,45,46,47,48,49,50,51,52,53,54]
Nano-based technologies	-A wide-range of formulations and combinations available -Physical parameters enable penetration into dense biofilm matrix -Can be coated onto dressings, bandages, sutures, drains -Reduced likelihood of resistance development	-Often effective only in combination with conventional antibiotics but not as stand-alone therapy	-Several commercial products based on nanomaterials available and in commercial use	[55,56,57,58,59,60,61,62,63,64,65,66,67,68,69,70,71,72,73,74,75,76,77,78]
Blue light therapy	-Effective against a wide range of pathogens -Reduced likelihood of resistance development -Ease of administration -Observable adverse effects to host cells minimal -In use for skin ailments such as acne	-Less effective against Gram positive pathogens; important given the polymicrobial nature of wound biofilms	-In vivo preclinical evidence supporting its use -No reports of clinical trials for use in chronic wound biofilms	[79,80,81,82,83,84,85,86,87,88,89,90,91,92,93]
Quorum sensing inhibitors	-Potential to prevent early stage biofilm formation -A wide-range of potential therapeutic molecules available	-Highly strain/species-specific -Toxicity to host cells -Efficacy in complex, in vivo models is reduced -Yet to gain a foothold in infection management	-In vivo preclinical evidence with mixed results -No reports of clinical trials for use in chronic wound biofilms	[94,95,96,97,98,99,100,101,102,103,104,105,106,107,108,109,110,111,112,113,114]
**Antimicrobial therapies that target the chronic wound biofilm microenvironment, indirectly affecting microbial growth and survival**				
Modulation of pH	-In principle, pH modifying agents are easy to administer onto the wound surface -Less likelihood of resistance development	-Fine-tuning pH in the wound bed is a difficult approach -pH variations have multiple effects on several factors -Effects of pH depend on wound-specific conditions; no universal strategy possible	-Largely in vitro evidence with varied results	[115,116,117,118,119,120,121,122,123]
Negative Pressure Wound Therapy (NPWT)	-Standard of care in wound management -Almost no likelihood of resistance development -Well-suited for use in combination with antiseptic instillation	-Likely to be effective only in combination with conventional antiseptics but not as stand-alone therapy	-Already in use for wound care, can be leveraged to manage wound infections with more clinical studies and evidence-based practice	[124,125,126,127,128,129]
Hyperbaric Oxygen Therapy (HBOT)	-Standard of care in wound management -Almost no likelihood of resistance development -Can have minimal adverse effects if delivered locally	-Cumbersome delivery mechanism; local delivery devices need to be evaluated -Likely to be effective only in combination with other therapies but not as stand-alone therapy	-Already in use for wound care, can be leveraged to manage wound infections with more clinical studies and evidence-based practice	[130,131,132,133,134,135,136,137,138]
Surfactants	-Can be used to coat dressings, sutures, bandages -Less likelihood of resistance development	-Likely to be effective only in conjunction with antibiotics	-FDA approved surfactant polymer dressing available and in use	[139,140,141]
Electrical and Electrochemical approaches	-Almost no likelihood of resistance development -Can be combined with other agents in wound dressings	-Likely to be effective only in combination with other therapeutics but not as stand-alone therapy -Mode of delivery may not convenient	-Few commercial products available	[142,143,144,145,146,147]
**Antimicrobial therapies that target bacteria and the chronic wound biofilm microenvironment, both directly and indirectly impacting microbial growth and survival**				
Probiotics	-An established mode of therapy for other medical conditions -Less toxicity and adverse effects likely -Less likelihood of resistance development	-Could be counterintuitive to administer bacteria to treat an infection, this notion has to be overcome -Mode of delivery needs to be developed	-Reasonable body of in vitro and in vivo evidence; no specific wound infection product available	[148,149,150,151,152,153,154,155,156,157,158,159,160,161,162,163,164,165,166]
Mesenchymal stem cells	-Harness the ability of the innate immune system -Less likelihood of resistance development -Can be administered via bioengineered skin grafts or dressings	-Likely to be effective when combined with antibiotics -Ethical considerations (particularly for parenteral administration)	-In vivo preclinical evidence promising -Needs robust clinical evaluation to take it forward, approvals for which are likely to be rigorous	[167,168,169,170,171,172,173,174,175,176,177,178,179,180,181,182]
